# An approach for unsupervised interaction clustering in human–robot co-work using spatiotemporal graph convolutional networks

**DOI:** 10.3389/frobt.2025.1545712

**Published:** 2025-10-01

**Authors:** Aaron Heuermann, Zied Ghrairi, Anton Zitnikov, Abdullah Al Noman, Klaus-Dieter Thoben

**Affiliations:** 1 Faculty 04: Production Engineering - Mechanical Engineering and Process Engineering, University of Bremen, Bremen, Germany; 2 ICT Applications for Production (IKAP), BIBA – Bremer Institut für Produktion und Logistik GmbH at the University of Bremen, Bremen, Germany

**Keywords:** human–robot interaction, human–robot co-work, interaction capturing, interaction modeling, spatiotemporal graph convolutional network, clustering

## Abstract

In this paper, we present an approach to cluster interaction forms in industrial human–robot co-work using spatiotemporal graph convolutional networks (STGCNs). Humans will increasingly work with robots in the future, whereas previously, humans worked side by side, hand in hand, or alone. The growing frequency of robotic and human–robot co-working applications and the requirement to increase flexibility affect the variety and variability of interactions between humans and robots, which can be observed at production workplaces. In this paper, we investigate the variety and variability of human–robot interactions in industrial co-work scenarios where full automation is impractical. To address the challenges of interaction modeling and clustering, we present an approach that utilizes STGCNs for interaction clustering. Data were collected from 12 realistic human–robot co-work scenarios using a high-accuracy tracking system. The approach identified 10 distinct interaction forms, revealing more granular interaction patterns than established taxonomies. These results support continuous, data-driven analysis of human–robot behavior and contribute to the development of more flexible, human-centered systems that are aligned with Industry 5.0.

## 1 Introduction

Humans will increasingly work with robots as time progresses, whereas previously, humans worked side by side, hand in hand, or alone, particularly in handicraft and industrial productions with small batch sizes, which are heavily impacted by demographic change and the associated shortage of skilled workers (Heuermann et al., 2024). For years, apprenticeship positions have remained unfilled, and the number of apprentices has steadily declined. Meanwhile, the workforce is aging, and increasing numbers of skilled workers are leaving the workforce due to age (Glück, 2022; Statista, 2022a; Statista, 2022b; Statista, 2022c). The shortage of qualified workers is also worsened because handcraft occupations are often not considered an adequate alternative to academic careers. Craft occupations are still underestimated with respect to the content, requirements, prospects, and technical progress at the corresponding workplaces (Mischler, 2017).

Automation offers a way out of the shortage of skilled workers, which is considered the most remarkable business risk by companies based on industry and handcraft, along with production costs. Ultimately, the lack of suitable skilled workers can lead to production stoppages (Statista, 2022d). However, in many workplaces currently facing a lack of skilled workers, full automation is impossible or only possible to a limited extent because they are too complex. Production workplaces are complex when the tasks to be performed are diverse, not entirely deterministic, uncertain, and time-varying (dynamic) (Krause and Gebhardt, 2018). A large variety of products and variants, which goes hand-in-hand with small batch sizes and order-specific production in handcraft, increases the complexity of production workplaces, especially regarding variety and variability. Thus, these jobs often require human cognitive and motor skills to cope with the complexity (TÜV AUSTRIA and Fraunhofer Austria Research, 2016; Müller et al., 2019; Petzoldt et al., 2021). Whereas , dexterity is needed, for example, when wiring electronic products, where cable positions cannot always be determined, creativity, innovation, and adaptability are often required for manual modification work.

Against this background, human–robot co-work—more commonly called human–robot collaboration—is essential for dealing with the complexity in production workplaces, demographic change, and the reduction of the shortage of skilled workers in industry and handcraft (Glück, 2022). In this context, humans and robots work together in production processes, flexibly sharing tasks and contributing their respective skills (Müller et al., 2019). On the one hand, robots can relieve the aging workforce from ergonomically unfavorable work tasks, and their performance can be maintained for longer. On the other hand, robots can at least partially compensate for missing junior staff in the workforce (Glück, 2022). As a result, human–robot co-work has the potential to preserve jobs and maintain the production capacity in high-wage countries such as Germany, where the skilled labor shortage is particularly acute (Glück, 2022). Moreover, robots can make production processes more humane and appealing by taking on repetitive or strenuous tasks. In this way, human–robot co-work supports greater human-centricity, which is a key element of the Industry 5.0 paradigm, alongside resilience and sustainability (European Commission, 2021). Industry 5.0 envisions a production process where technology augments human capabilities rather than replacing them. Therefore, an increasing number of people will come into contact and interact with robots at their workplaces. This assumption is reinforced by the continuously increasing sales figures of collaborative robots (IFR, 2021). The complexity also affects the variety and variability of interactions between humans and robots at the considered production workplaces.

Industrial human–robot co-work distinguishes among different interaction forms, particularly coexistence, sequential and parallel cooperation, and (responsive) collaboration (Behrens, 2019; IFR, 2024). These interaction forms can be distinguished based on space, time, and physical contact characteristics (Spillner, 2014; Behrens, 2019). Although distinctions of interaction forms, such as the ones above, are established in industry and handcraft, these taxonomies are essentially theoretically derived and not unified (Behrens et al., 2015; Spillner, 2014; Bauer et al., 2016; Onnasch et al., 2016; Behrens, 2019; Petzoldt et al., 2021; Glück, 2022). A data-based investigation of the actual interaction behavior between humans and robots in production processes has not been conducted yet to validate the theoretical considerations. Instead, various studies and surveys attempt to assign real use cases exclusively to one of the forms (Bauer et al., 2016).

Human–robot co-work is useful where full automation is impossible or not profitable due to the required flexibility and capabilities (Müller et al., 2019). However, this means that production processes, task divisions, and, thus, the interactions between humans and robots change continuously or at least regularly during operation. Consequently, a continuous investigation of the interactions is required. Due to expected changes during operation, such as the changes due to wear and tear of the technical systems and different product variants or generations, regular (and occasion-related) reviews of the respective human–robot co-work must be carried out (Glück, 2022). However, these often require great manual effort and are different from continuous system monitoring, which would be needed in the context of continuously changing human–robot co-work at complex production workplaces.

For a long time, continuous observation and investigation of the interaction behavior of humans and robots in production processes was inconceivable because manual recording and evaluation required great effort. Advances in data acquisition and processing, the higher availability of data, and the methods or tools for data analysis are now making more automated approaches possible. Thus, computational ethology promises new insights and a better understanding of (human) behavior through automatic data collection and data-based investigations (Mobbs et al., 2021). In addition, data-based activity and context recognition are already being used to investigate and gain insights into manual industrial processes (Feldhorst, 2018). Similar approaches also promise insights into the co-work of humans and robots in industry and handcraft and into the actual interaction behavior in ongoing operations.

Therefore, in this paper, we aim to better understand the variety and variability of interactions between humans and robots in complex production workplaces by introducing an unsupervised approach for interaction clustering in human–robot co-work using spatiotemporal graph convolutional networks (STGCNs). Furthermore, the approach’s feasibility is investigated using a novel dataset collected in realistic human–robot co-work scenarios using a high-accuracy tracking system.

In summary, this paper’s contributions are as follows:

• An unsupervised approach for automatically clustering human–robot interactions using STGCNs combined with k-means clustering.
• A novel dataset covering 12 realistic human–robot co-work scenarios collected using a high-accuracy tracking system.
• A feasibility confirmation of the proposed approach on the collected dataset, providing qualitative evidence of its effectiveness in capturing and distinguishing various human–robot interaction forms.
• Empirical evidence that the interaction behavior in human–robot co-work is more diverse than that suggested by established taxonomies (identifying 10 distinct interaction forms instead of four).
• Supporting human-centered robotics aligns with the Industry 5.0 vision, enabling more flexible and intuitive human–robot co-work.

## 2 Background and related work

### 2.1 Capturing interactions in human–robot co-work

Robots can only replace human interaction partners and become colleagues in the workplace if they possess interaction skills similar to those of humans (Müller et al., 2019). From a psychological and sociological perspective, interaction is characterized by mutual influence and interdependency between the agents (Becker-Beck, 1997; Israel, 2003). A human actor influences the processes at the production workplace through his actions, just as his actions are influenced by the production workplace and the actions of the other actors, for example, robots (Israel, 2003). Human–robot interaction refers to “all actions and behavior between humans and robots” (Spillner, 2014) and consequently includes human–robot co-work.

Human–robot co-work refers to humans working or interacting with robots on the same object, often with varying physical proximity and temporal coordination. This work-sharing between humans and robots and the physical interaction between humans and robots in a work context is more commonly referred to as human–robot collaboration (Behrens, 2019). At the same time, collaboration refers to “hand-in-hand” co-work to achieve a common goal that requires physical contact between humans and robots (Behrens, 2019). Therefore, the term collaboration is used to describe the overarching area and refers to an interaction form that is subordinate to this area. To avoid confusion, we use the more neutral term human–robot co-work in this paper.

Interaction forms in industrial and handcraft contexts are commonly categorized into coexistence, sequential cooperation, parallel cooperation, and (responsive) collaboration, which are based on spatial, temporal, and physical contact characteristics (Behrens, 2019; IFR, 2024). These established taxonomies are predominantly theory-driven, not standardized, and lack empirical validation (Behrens et al., 2015; Spillner, 2014; Bauer et al., 2016; Onnasch et al., 2016; Behrens, 2019; Petzoldt et al., 2021; Glück, 2022). A data-based investigation of the actual interaction behavior between humans and robots in production processes has not been conducted yet to validate the theoretical considerations. Instead, various studies and surveys attempt to assign real use cases exclusively to one of the forms (Bauer et al., 2016).

Due to the ongoing robotization, the variety of observable applications of collaborative robots in production workplaces is also growing. Now, there are applications for many manufacturing processes and assembly functions, including handling. However, most industry and handcraft applications have involved humans and robots working together less closely (El Zaatari et al., 2019; IFR, 2024). This growing variability calls for data-driven approaches to model and classify interactions more systematically. Robot behavior can be described through internal data (e.g., joint angles and velocities), whereas human behavior must often be captured via motion tracking or indoor localization systems (Feldhorst, 2018).

Interactions between humans and robots can be studied by fusing these data and looking at them together. Although the recognition of human activities in industrial processes has already been considered many times (Feldhorst, 2018), more studies regarding the interaction behavior of humans and robots when working together in production processes are needed. Although data on humans and robots are collected together in some cases, these have been used particularly for distance calculation in the context of continuous speed, separation monitoring, and collision detection (Müller et al., 2019). Furthermore, in various works, data about humans and robots are fused to enable flexible hand-to-hand transfers but not to improve interactions across tasks (Maeda et al., 2017; Tang et al., 2020). Other authors focus on building accurate digital human models using real-time motion-tracking systems, such as those for ergonomics or safety purposes (Tuli and Manns, 2020). Tuli and Manns (2020) captured human behavior during a collaborative assembly process and derived tasks, which included human motion sequences, such as reach, join, apply force, and release. Heuermann et al. (2024) presented a sensor-data-based approach for automatic, continuous capturing of the variety in human–robot interactions and an interaction modeling approach based on spatiotemporal graphs (Heuermann et al., 2024). Vogt et al. (2017) introduced an imitation learning approach for human–robot interactions from human–human demonstrations.

In summary, although conceptual interaction models provide a valuable foundation, a more flexible and granular understanding of human–robot co-work is needed: one that is grounded in empirical data and can account for the variety and variability of interactions observed in industrial and handcraft production settings.

### 2.2 Modeling human–robot interactions with graphs

Physical human–robot interaction is the interrelated spatiotemporal behavior of humans and robots, and can be characterized by space, time, and physical contact (Spillner, 2014; Behrens, 2019). Graphs are an option for abstract representation and investigation of spatial dependencies and interactions (Schmidt, 2023). A graph consists of nodes and edges (Tittmann, 2019; Schmidt, 2023). Graphs are already widely used in modeling actions and interactions at production workplaces. Many applications can be assigned to human activity recognition (HAR) and ergonomics analysis. For this purpose, human posture is often determined using video or image data, and the skeleton is modeled as a graph. Whereas nodes in the graph represent the joints in the body, the edges correspond to the bones that connect the joints (Yan et al., 2018).

The actions of individual actors and the interactions of several actors can be modeled using graphs. One approach for describing spatial relationships between multiple actors are interaction meshes (Vogt et al., 2017). Interaction meshes were introduced in computer animation for realistic movements of characters during interactions with nearby body parts and objects (Ho et al., 2010; Vogt, 2018). However, robotics also uses interaction networks, especially for human–robot interaction and path planning (Vogt et al., 2017; El Zaatari et al., 2019). As the computational complexity increases significantly with the number of nodes and edges, the number of nodes and edges considered should be minimized. To this end, Vogt et al. (2017) and Vogt (2018) presented a correlation-based approach, which uses the correlation between the movements of different nodes to eliminate and weigh edges, and a context-based approach, which includes additional context information in addition to the correlation between node movements. Both approaches result in less densely connected interaction networks, which reduce the computational effort and enable the short response times required in robotics. The approaches do not require deriving complete human models from video or image data or an extensive system of markers or trackers. The interaction networks can be created using only the nodes involved in the interaction. This means that the approaches can also be combined with indoor localization or motion-tracking systems that do not have many markers or trackers. Vogt et al. (2017) recognized that human–robot interactions can benefit significantly from representations using spatial graphs. A mere mapping of spatial relationships and dependencies is insufficient for investigating individual actors’ actions or the interactions of multiple actors. Therefore, sequences of these are considered rather than individual spatial graphs. For this purpose, a spatial graph is generated for each video frame, point in time in time-series data, or state in a process. Temporal dependencies can then be mapped through transition probabilities from one state to another or recurring patterns that represent the spatiotemporal behavior of the actors.

### 2.3 Spatiotemporal graph convolutional networks

If there are no explicit rules or regulations for programming a machine to solve a task, machine learning from data and experience is an alternative (Paaß and Hecker, 2020). For instance, it can be utilized when previously unknown relationships, dependencies, or patterns in data need to be identified and investigated. Machine learning approaches, particularly deep learning, are increasingly used to examine temporal and spatial dependencies and correlations in spatiotemporal graphs. Zeghina et al. (2024) provided a more comprehensive overview of different approaches and applications of deep learning based on spatiotemporal graphs. In addition to STGCNs, other approaches for modeling human–robot interactions include recurrent architectures such as long short-term memory (LSTM) networks and temporal convolutional networks (TCNs), attention-based models such as transformers or graph attention networks (GAT), or their combinations, and classical methods such as hidden Markov models. Unlike sequential models such as LSTMs or TCNs, which primarily capture temporal patterns, STGCNs explicitly model spatial relationships by representing entities such as humans, robots, and objects as nodes in a graph. This allows the network to jointly model the spatial and temporal aspects of multi-agent interactions in an interpretable, scalable structure. In contrast to other approaches, STGCNs inherently scale to multi-agent and multi-object scenarios, making them suitable for modeling the varied interactions found in human–robot co-work.

STGCNs are deep machine learning models designed to handle data with spatial and temporal dependencies by integrating graph convolutional layers and temporal convolutional layers through spatio-convolutional blocks (Yu et al., 2017). Whereas the spatial behaviors or relationships are modeled in STGCNs using graph convolutional networks (GCNs), temporal dynamics are modeled using approaches such as convolutions (Conv1D), gated recurrent units (GRU), or temporal attention.

STGCNs show promising results on graph-structured data in various applications, such as traffic forecasting (Yu et al., 2017), human action recognition (HAR) (Yan et al., 2018), and social network analysis. Yan et al. (2018) performed human pose estimation on videos, constructed a spatiotemporal graph on the skeleton sequence, and classified human activities using an STGCN output (Yan et al., 2018). In addition, Liu et al. (2024) extracted skeleton sequences from videos and used them in a graph convolutional neural network with temporal attention to classify and predict human interaction behavior for human–robot co-work. However, only the human is represented as a graph, not the interaction scenario, including the robot and the object (Liu et al., 2024). Xing and Burschka (2024) used an architecture combining graph and temporal convolutions to understand the spatiotemporal relations in human–object interaction better, using extracted graphs from video data (Xing and Burschka, 2024).

Several recent studies have applied STGCNs or related graph-based models for human activity recognition, but they differ significantly from the approach presented in this work. For instance, Yan et al. (2018) and Liu et al. (2024) focused primarily on human skeleton-based action recognition and represented only the human as a graph, without modeling the robot or the objects involved in the interaction. Similarly, Xing and Burschka (2024) analyzed human–object interactions using video-derived graphs, but they do not extend this to full human–robot–object interaction scenarios. Moreover, these approaches typically rely on supervised learning with predefined activity labels and are often limited to specific tasks. In contrast, in this paper, we propose an unsupervised approach that models realistic human–robot–object interactions using tracking data collected from various human–robot co-work scenarios. This enables a more general and data-driven understanding of the interaction behavior, beyond the predefined activity classes or human-only modeling.

## 3 Materials and methods

**FIGURE 1:**
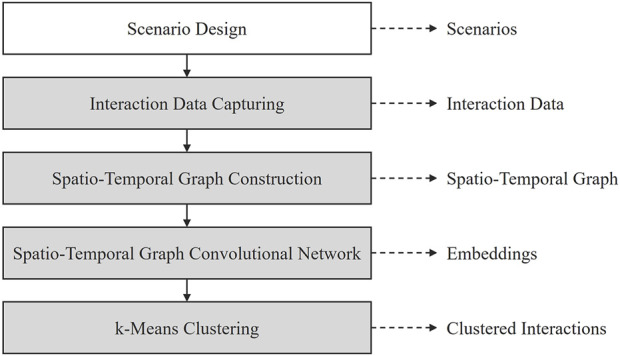
Approach for unsupervised interaction clustering in human–robot co-work using spatiotemporal graph convolutional networks.

In this section, we introduce the proposed approach for unsupervised clustering of interactions in industrial human–robot co-work using STGCNs. The approach includes capturing the interaction data (section 3.2) in previously designed human–robot co-work scenarios (section 3.1), constructing a graph-based representation of the interactions (section 3.3), the STGCN for feature learning, and unsupervised interaction clustering (section 3.4). Figure 1 provides an overview of this approach and its main components.

### 3.1 Scenario design for data collection

**FIGURE 2:**
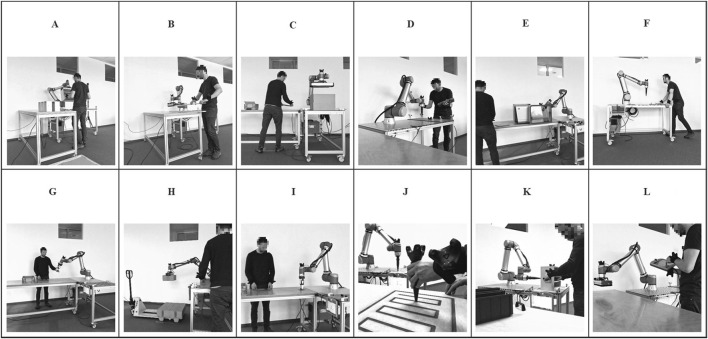
Human–robot co-work scenarios for data collection. **(A)** Assembly. **(B)** Co-Manipulation. **(C)** Commissioning. **(D)** Gluing. **(E)** Machine Tending. **(F)** Moving. **(G)** Overhanding. **(H)** Palletizing. **(I)** Pick and Place. **(J)** Sanding. **(K)** Screwing. **(L)** Teaching.

The present investigation aims to consider and capture the broadest possible variety of human–robot interactions in industrial human–robot co-work. Therefore, the authors decided against data collection from industrial environments. Instead, data were collected from realistic yet simplified scenarios replicating key characteristics of industrial human–robot co-work without the full complexity of production environments. These scenarios were selected and designed to cover the broadest possible spectrum of human–robot interactions and applications observed in industrial human–robot co-work, encompassing physical and supervisory interactions. Therefore, the scenarios were selected to cover the various assembly functions according to Lotter (2012) and the functions of object handling. Furthermore, the selection criteria included the coverage of established interaction forms across key characteristics (spatial proximity, temporal coordination, physical contact, and task structure) in alignment with common industrial taxonomies such as coexistence, sequential cooperation, parallel cooperation, and collaboration. Figure 2 shows the 12 resulting scenarios, which is followed by their descriptions.

All scenarios are implemented using a Universal Robot UR10e, which is equipped with different end-effectors depending on the scenario and mounted on a movable workbench. In most scenarios, another (movable) workbench serves as a workplace for humans.

A – Assembly: in this scenario, the human and robot work together to assemble a wooden box with drawers. At the beginning of the process, the human and the robot work independently beside each other. While the robot executes a simple pick and place task repeatedly, the human picks the drawers from a pallet and places them in his workplace. As soon as the human is ready for the next step in the process, the robot’s assistance is requested by pushing a button. The robot grips a wooden box from a predefined place with a vacuum area gripper and provides it to the human at an ergonomic working height. The human assembles the product by putting the drawers into the wooden box. After the assembly, the robot rotates the box before the human for a final visual quality control before placing it at a predefined place.
B – Co-manipulation: the human and the robot work hand-in-hand in this scenario to handle long wooden planks. As soon as the human is ready, the human requests robot assistance by pressing a button. Then, the human and the robot simultaneously grasp a plank at opposite ends (the robot with a vacuum area gripper), lift it off the stack, move it, and place it on another stack.
C – Commissioning: during commissioning, the human packs products in an envelope at his workstation and places it at a predefined transfer position. While the robot picks the envelope from this position with a vacuum area gripper and puts it in a box, the human prepares the next envelope. The robot pauses when the box is filled, and the human closes it and replaces it.
D – Gluing: in this scenario, the robot is equipped with a 3D-printed end-effector, which simulates a glue dispenser unit. The human places and fixes a part to be glued on the robot workbench and starts the robot gluing process by pushing a button. Then, the robot moves along the gluing trajectory, which the human had taught beforehand using the robot’s teach pendant combined with hand guidance. The human waits and exchanges the part when the robot has finished gluing. From the interaction perspective, this gluing application is also comparable to welding applications.
E – Machine tending: in the machine tending scenario, the robot operates, loads, and unloads another machine, such as the Formlabs Cure L for post-curing SLA 3D-printed parts. Therefore, the robot opens the machine door, picks a 3D-printed part from a magazine, and places it inside the machine. Then, the robot closes the door and waits until the machine process is finished before replacing the 3D-printed part with another one. The robot uses a clamping gripper to handle the machine doors and the parts. The human is responsible for exchanging the part magazine and restarting the robot process after changing. The human works separately on an independent production process while the robot performs the machine tending.
F – Moving: robots are increasingly being used in varying workplaces. Therefore, humans must move the passive (turned off) robot to its next workplace. In this scenario, the operator pushes the robot on the movable workbench to a predefined position while it is turned off and not moving on its own.
G – Overhanding: in this scenario, the human places and fixes a magazine with 12 parts on the robot workbench, walks to the human workplace, and starts the robot by pushing a button. The robot iteratively picks a part from the magazine using a clamping gripper, moves it to a changing overhanding position close to the human workplace, and hands it over directly into the human hand. While the human proceeds with the part, the robot picks the next part from the magazine.
H – Palletizing: during palletizing, the human uses a pallet truck to provide a pallet for the robot and starts the robot’s palletizing process. The human then places packages at a predefined transfer position. From there, the robot picks the packages using a vacuum area gripper and stacks four packages in a layer on the pallet. When the palletizing is complete, the human exchanges the pallet with the pallet truck.
I – Pick and place: the pick and place scenario is mainly similar to the overhanding scenario (G). Instead of directly handing the part to the human, the robot safely places the part at a predefined place. The human takes the part from there when the robot has moved away.
J – Sanding: the sanding scenario includes teaching by demonstration. The human places and fixes a part to be sanded on the robot workbench, walks to the human workplace, and starts teaching the robot by demonstration. Therefore, the human uses a 3D-printed teaching tool, which is equipped with an object tracker, and moves it over an identical part to generate the sanding trajectory. The robot follows this trajectory on its part with a delay. A 3D-printed end-effector is used in this scenario instead of a sanding machine, but the robot’s behavior is comparable. Furthermore, this scenario is similar to painting applications with teaching by demonstration.
K – Screwing: in this scenario, the robot is equipped with a 3D-printed end-effector, which simulates a screw driver unit. At the beginning, the human places a wooden box at a predefined position on the robot’s workbench and starts the robot by pushing a button. While the robot approaches four screw positions on the back, the human attaches handles to the left and right sides of the box, each with two screws. The human then rotates the box first by 90° and then by 180° so that the robot can tighten the screws on the handles to a specified torque.
L – Teaching: teaching is not a separate scenario but involves real robot teaching and programming. While implementing the scenarios described above, data were collected during the robot’s programming via the teach pendant and hand guidance.

In summary, the 12 selected scenarios represent a comprehensive and exhaustive set of human–robot co-work situations that reflect real industrial applications (e.g., assembly, sanding, and screwing) while remaining feasible for controlled laboratory data collection. They cover a wide range of interaction forms, including physical collaboration (e.g., co-manipulation and overhanding), sequential and parallel cooperation (e.g., commissioning and palletizing), and supervisory interactions (e.g., teaching). The scenarios vary in spatial proximity, temporal coordination, and physical contact, and they include varying task complexities and human–robot involvement. Together, the scenarios provide a robust foundation for capturing the variety and variability of human–robot interactions in industrial human–robot co-work.

### 3.2 Interaction data capturing

The interrelated spatiotemporal behavior can be captured using an accurate indoor localization system. Most available indoor localization systems based on Wi-Fi, Bluetooth, or RFID (meter accuracy), UWB (decimeter accuracy), or ultrasonic (centimeter accuracy) are too inaccurate to capture fine interaction sequences (Obeidat et al., 2021).

The VIVE Lighthouse tracking system, based on infrared (IR), showed a submillimeter or a few millimeter accuracy, reproducibility (Bauer et al., 2021; Heuermann et al., 2024), and low latency in various investigations. It has already been used for robotics several times in research (Tuli and Manns, 2020; Tuli et al., 2022; Heuermann et al., 2024) and industry, such as Wandelbots or Nordbo Robotics. Wandelbots and Nordbo Robotics provide no-code programming and teach-by-demonstration solutions for robots based on the Lighthouse tracking system. This indoor localization system consists of at least one IR transmitter, a so-called Lighthouse base station, and at least one tracker. The IR base stations send a dense grid of IR laser beams into the room at millisecond intervals. The IR beams strike sensors (photodiodes) placed on the trackers. Based on the measurement data, the current position and movement of the trackers in space can be triangulated (Heuermann et al., 2024).

**FIGURE 3:**
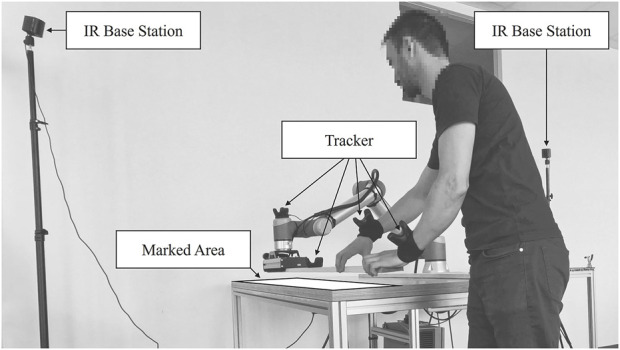
Setup and tracker placement for capturing human–robot interactions.

Typically, hands are directly involved in co-work at complex production workplaces. Human and robot arms use their hands, a gripper, or an end-effector to manipulate or handle objects. Thus, the human and robot hands and, if applicable, the manipulated or handled objects have to be considered when capturing human–robot interactions. Subsequently, trackers are required to capture the following positions. One tracker has to be fixed close to the robot tool-center-point to obtain the robot position $P_{R}$ . Two additional trackers are needed on the human’s wrists to capture the left-hand position $P_{HL}$ and the right-hand position $P_{HR}$ . In production workplaces, humans and robots usually work on parts (Conti et al., 2020), products, and tools—generally on or with objects. Tracking these objects can help understand whether humans and robots share a common goal. Attaching a tracker to an object can capture its position*.* However, it is not always possible to add trackers to objects, such as when they are too small, change too frequently, or a tracker would hinder the foreseen object manipulation. In such cases, static virtual objects can be set in advance. For this purpose, the object’s picking, placing, handover, or processing areas are marked with a tracker, and the positions are memorized. Whenever humans or robots approach these positions or areas later in the production process, it is equivalent to approaching the object. This concept can also mark work areas and teach pendants or human–machine interfaces (HMIs) to capture interactions between the robot and the operator. Figure 3 shows the setup, including the tracker placement for capturing human–robot interactions using the Lighthouse tracking system.

### 3.3 Spatiotemporal graph construction

To enable learning from the spatial and temporal dependencies or behaviors in human–robot interactions, each interaction instance is represented as a spatiotemporal graph. This section defines the graph structure used as input for the STGCN and explains the encoding of the spatial and temporal dependencies in the data.

**FIGURE 4:**
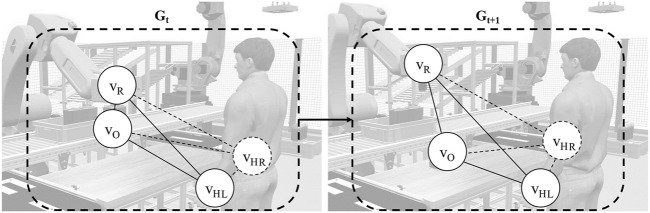
Human–robot interaction modeled as a spatiotemporal graph.

For every point in time ***t*** in the production process or each dataset, the current state of the interaction among humans, robots, and objects can be modeled as a three-dimensional spatial graph ***G***_***t***_. Figure 4 illustrates human–robot interaction as a sequence of spatial graphs or a spatiotemporal graph.

A graph ***G***_***t***_ ***= (V, E, W)*** consists of the following:

**Nodes (*V*):** a set of nodes or vertices, which represent entities in the graph.

$$V=\left\{v_{1},v_{2},\ldots ,v_{n}\right\},where\;v_{i}\in V.$$

At a given moment, the robot holds a part while the human stands nearby waiting. The system tracks the entities, which are the robot, the human’s left and right hands, and the handled object. Each is represented as a node ***v*** in the graph. Subsequently, the nodes ***v***_***R***_, ***v***_***HL***_, ***v***_***HR***_, and ***v***_***O***_ exist in the graph. ***|V|*** denotes the number of nodes in the graph.

The nodes ***V*** are represented as a tensor with the shape ***[N, T, F]*,** where ***N*** is the number of vertices or nodes, ***T*** is the number of time steps, and ***F*** is the number of input features. Each node is represented by a feature array ***F*** that encodes the node type and its spatial relationship to other nodes. The features $is_{R},is_{HL},is_{HR},$ and $is_{O}$ are binary indicators denoting the node type. If the considered node represents the robot, the feature $is_{R}$ is set to 1, and the others are set to 0. The remaining features represent the Euclidean distances d from the current node to all other nodes in the graph at the same time step.

$$F_{t}=\left[is_{R},is_{HL},is_{HR},is_{O},d_{ij,t},d_{ij+1,t},d_{ij+2,t}\right].$$

For instance, if at the given moment the robot is 1,000 mm from the human’s left hand, 900 mm from the right hand, and 250 mm from the object, the robot node feature array is as follows:

$$F_{R,t}=\left[1,0,0,0,1000,900,250\right].$$

Further features could be added as an extension to map further interactions, such as micro patterns or the proximity of the human hands to buttons.

**Edges (E):** A set of edges represent the connections or relationships between the nodes. Each edge e∈E is a pair of nodes. In the present application, the spatial graph represents the spatial dependencies of the nodes in the three-dimensional space as Euclidean distances in millimeters. Subsequently, the graph is an undirected graph, where the edges have no direction. Furthermore, it is a fully connected graph.

$$e=\left\{u,v\right\},u,v\in V.$$

The node connections can also be represented by an adjacency matrix ***A***.

**Weights (W):** A weight function assigns a numerical value (weight) to each edge. As described, the spatial graph represents the spatial dependencies of the nodes in the three-dimensional space as Euclidean distances ***d*** in the present application.

$$w\left(u,v\right)=d\left(u,v\right)=\sqrt{{\left(u_{x}-v_{x}\right)}^{2}+{\left(u_{y}-v_{y}\right)}^{2}+{\left(u_{z}-v_{z}\right)}^{2}\;}.$$

This graph-based representation is the input for the STGCN described below. It models spatial relationships in a structured, flexible way that adapts to varying interaction setups and is easily generalizable across various human–robot co-work scenarios. By encoding each actor’s identity and spatial configuration, the graph enables the model to capture subtle variations in how humans, robots, and objects relate to one another even within similar tasks. This structured input enhances the model’s ability to distinguish nuanced interaction patterns and supports interpretable analysis of physical interactions.

### 3.4 Spatiotemporal graph convolutional network-based clustering

**FIGURE 5:**
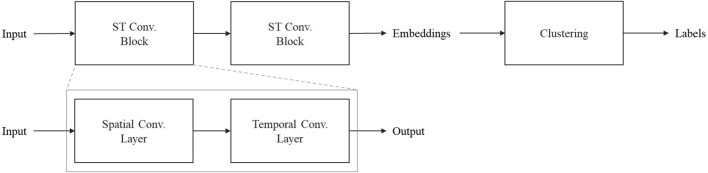
Architecture of spatiotemporal graph convolutional network-based clustering.

The aim was to identify distinct interaction forms in human–robot co-work. Therefore, the STGCN is combined with a clustering algorithm. While the STGCN is a promising approach for modeling complex spatial and temporal dependencies or behaviors in multivariate time-series data, the model embeddings can be used as features in an unsupervised clustering algorithm, such as k-means, to identify distinct interaction forms and label the collected data. This architecture is shown in Figure 5.

The proposed STGCN architecture consists mainly of two sequential spatiotemporal convolution blocks (ST conv. block) and is implemented using PyTorch. Each spatiotemporal convolution block consists of a spatial convolution layer followed by a temporal convolution layer.

The graph convolutional operator from the study by Kipf and Welling (2016) implemented in torch_geometric.nn as conv.GCNConv (PyTorch-geometric, o.J.) is used as a spatial convolutional layer. For a thorough description of the functioning and algorithms, see Kipf and Welling (2016) and the PyTorch-geometric documentation. A Conv1d is used as temporal convolution, which “applies a 1D convolution over an input signal composed of several input planes” (PyTorch, o.J.).

The STGCN has seven input and output channels, 64 hidden channels, and a kernel size three for the temporal convolution. Consequently, the spatial convolutional layer in the initial spatiotemporal convolutional block applies a graph convolutional network (GCN) operation to the input graph with an input feature size of seven and an output feature size of 64. The following temporal 1D convolutional layer has 64 input and output channels, a kernel size of three (looking at three time steps at a time), a stride of one (moving step-by-step), and a padding of 1, which maintains the same output length as the input. In the second spatiotemporal convolutional block, another GCN operation is applied in the spatial convolutional layer, reducing the feature size from 64 to seven. Finally, another temporal 1D convolution is carried out with seven input and output channels. An overview of the used layer configuration is shown in Table 1. The ReLU (rectified linear unit) activation function is applied after each convolutional layer.

**TABLE 1:** Layer configuration.

Block	Layer	Type	Input channels	Output channels	Notes
1	1	GCNConv	7	64	Graph convolution
	2	Conv1D	64	64	Kernel = 3, stride = 1, and padding = 1
2	3	GCNConv	64	7	Graph convolution
	4	Conv1D	7	7	Kernel = 3, stride = 1, and padding = 1

For training, a maximum of 150 epochs is defined, and an early stopping mechanism is implemented to prevent overfitting and save computational resources when further improvement is unlikely. The early stopping is triggered after five consecutive epochs without improvement.

An Adam optimizer is used for training the model with an initial learning rate of 0.001 and a weight decay of 1e-4 to prevent overfitting by penalizing large weights. It is used in combination with a learning rate scheduler (ReduceLROnPlateau), which halves (factor is set to 0.5) the learning rate after three (patience is set to three) consecutive epochs without loss improvement. The Huber loss is used as the loss function, which combines the strengths of the MAE loss (more robust to outliers) and the MSE loss (sensitive to minor deviations).

The STGCN outputs its embeddings as output features for each node and time step. These embeddings are the input features for the subsequent clustering to identify distinct interaction forms. K-means is used in the proposed architecture as the clustering algorithm.

K-means clustering is an unsupervised algorithm that groups unlabeled data into clusters. It is a centroid-based algorithm, which assigns each data point to the cluster with the closest centroid (Joshi, 2023). The number of clusters *k* has to be defined before the model training. To determine the optimal number of clusters, k-means clustering is carried out for k = 2 to k = 20, and performance indicators, such as the sum of squared errors (SSEs), the Davies–Bouldin index (DBI), and the Calinski–Harabasz index (CHI), are calculated. The cluster assignments for all data points carried out during the final clustering with optimal *k* are added as labels to the data.

## 4 Analysis and results

### 4.1 Dataset and data preparation

For generating the dataset, the 12 human–robot co-work scenarios were executed multiple times while collecting tracking data. A total of 1.32 million data points from up to four trackers were collected in a fixed interval of 10 milliseconds. Subsequently, 13,280 s or 3.69 h of data were collected while executing the scenarios. To accelerate the training and identify temporal relationships over a larger interval, every 10 data points were taken, so the interval was 100 milliseconds. All data were standardized using the standard scalar, and six subsets were built from the dataset. Each subset contained data from all the scenarios. Whereas five subsets were used for training the STGCN and clustering, one subset was reserved for testing.

A rolling window approach was used to generate batches from the time-series tracking data to prepare the data for training. Each batch consisted of 40 consecutive time steps (i.e., a sequence of 4 s sampled at 100 ms intervals), and the window was moved forward by 10 time steps (1 s) to generate the next batch. This resulted in a 75% overlap between successive batches. The use of overlapping windows allows the model to capture patterns across different parts of the sequence and ensures that relevant temporal dependencies are preserved throughout training. Sliding window approaches are widely used in pattern recognition and ensure patterns are learned and recognized without knowing their locations in the data. Each batch includes four nodes (human left hand, human right hand, robot, and object) and is used as one training example. A total of 11,257 batches were generated from the dataset, and they were used for training in each epoch.

### 4.2 Spatiotemporal graph convolutional network

**FIGURE 6:**
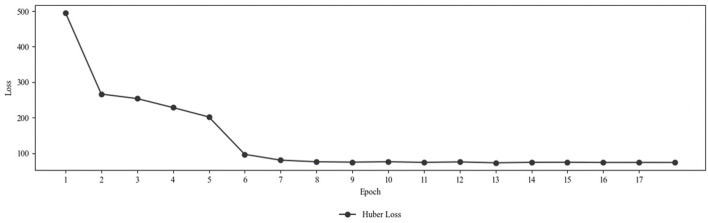
Huber loss progression over the training epochs.

The STGCN was implemented with the presented architecture in Python using the PyTorch library and supported GPU acceleration via CUDA for efficient computation. The training took between 927.76 s and 956.49 s per epoch. On average, the time taken was 934.42 s or 15.57 min. Figure 6 shows the progression of the Huber loss over the training epochs. The loss decreased significantly from 495.77 at epoch 1 to 96.75 at epoch 6. After epoch 6, improvements became smaller, with the loss decreasing gradually to 72.89 by epoch 13. Subsequent epochs show a slight increase or stagnation in loss, indicating that the model may be nearing convergence. Therefore, early stopping was triggered after epoch 17, and the training ended.

After training, all batches were re-entered into the model, and the resulting embeddings were saved for clustering. Due to the overlapping batches, 445,468 embeddings were generated from 112,342 data points for 4 x 7 or 28 output features. Therefore, embeddings that could be assigned to the same data points were aggregated using the mean method, resulting in 112,342 data points again.

### 4.3 Clustering

**FIGURE 7:**
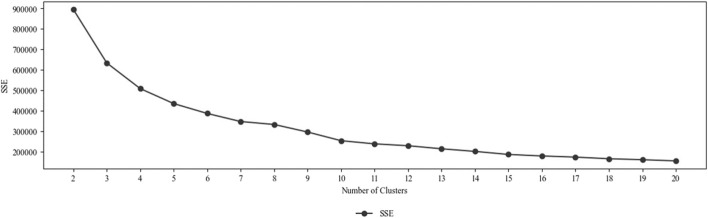
Sum of squared errors depending on the number of clusters.

A k-means clustering was applied on the embeddings to identify distinct interaction forms. To determine the optimal number of clusters, we evaluated k-means clustering results across values of k from 2 to 20 using standard validation metrics, namely, the elbow method (based on the SSEs), DBI, and CHI. Figure 7 shows the SSEs depending on the number of clusters.

According to the elbow method (knee locator) and the mentioned performance indicators, 10 is the optimal number of clusters. A total of 10 interaction forms can be distinguished in the considered human–robot co-work scenarios. This is supported by DBI (1.04) and CHI (50,766.61) values. The SSEs is 230,013.85. The final clustering was carried out for 10 clusters (k = 10). The cluster assignments for all data points carried out during the final clustering were added as labels to the data.

**FIGURE 8:**
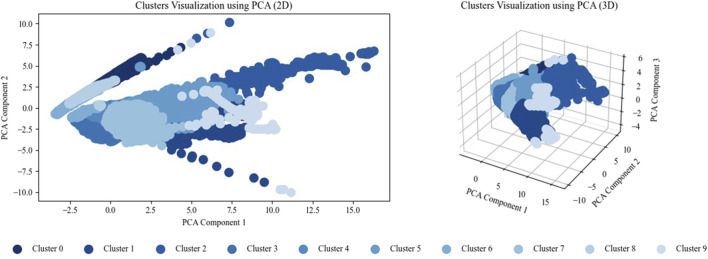
Cluster visualization using two-component (left) and three-component (right) PCAs.

Principal component analysis (PCA) is widely used in data science and machine learning for reducing high-dimensional data to two or three dimensions, such as for visualization. Whereas the PCA with two components explains 78.06% of the variance, 88.81% is explained with three components. The clusters are visualized using two-component and three-component PCAs, as shown in Figure 8.

As the proposed approach is unsupervised and applied to a novel dataset of unlabeled human–robot interactions, direct comparison with (supervised) established models is not feasible. No benchmark dataset offers the same structure, agent configuration (human, robot, and object), or labeling scheme used in this paper. Additionally, ground truth labels for interaction forms do not exist, which limits the applicability of accuracy-based metrics. Instead, we compare our approach to a feature-engineered baseline using standard clustering metrics (SSEs, DBI, and CHI) to estimate its relative performance, expressiveness, and clustering quality. Therefore, a k-means clustering was performed on the same dataset with engineered and calculated features. K-means is selected for comparison due to its compatibility with the continuous, high-dimensional feature space. K-means is widely used in machine learning contexts for clustering embeddings, as it assumes Euclidian distance between points and supports controlled selection of the number of clusters (k), which enables a consistent and interpretable comparison. In contrast, DBSCAN is a density-based clustering algorithm that relies on parameters such as neighborhood radius and minimum points, which are difficult to tune in high-dimensional spaces. K-means offered a more stable, interpretable, and computationally efficient baseline for evaluating the relative clustering performance of our proposed approach.

The Euclidean distances among the robot, the human’s left and right hands, and the object were calculated as features to cover the spatial dependencies. Time-series features, namely, lag, rolling statistics, and Fourier transforms, were built to cover the temporal dependencies in the features. The 10th previous value for each spatial feature was used as a lag feature. However, the lag features were removed due to significant correlations with the spatial features. The rolling mean and standard deviation features were calculated over a window of size 10 for all spatial features. Fourier transformations decompose the time-series data into an amplitude feature and a frequency feature for all spatial features. In this clustering, the elbow method (knee locator) suggested 11 clusters, but on comparing the SSE, DBI, and CHI, it was found that 11 clusters lead to a slightly lower SSE but to worse DBI and CHI values (SSE: 993,311.39, DBI: 1.34, and CHI: 16,101.37) than 10 clusters (SSE: 1,030,779.74, DBI: 1.33, and CHI: 16,555.66). Therefore, the final clustering was performed with k = 10 to improve the comparison with the STGCN-based clustering with the same number of clusters. Table 2 compares the metrics of the STGCN-based clustering (Clustering STGCN) and the clustering based on the engineered features (Clustering FE).

**TABLE 2:** Clustering performance comparison.

Approach	Number of clusters (k)	SSE	DBI	CHI
Clustering STGCN	10	230,013.85	1.04	50,766.61
Clustering FE	10	1,030,779.74	1.33	16,555.66

**FIGURE 9:**
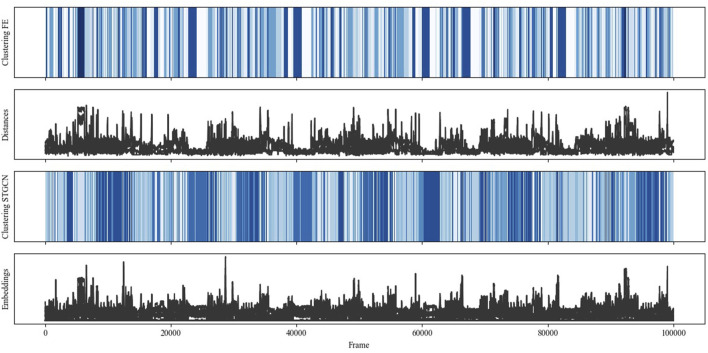
Comparing embeddings and STGCN-based clustering with distance features and clustering based on feature engineered (FE) data.

For a qualitative evaluation, Figure 9 contrasts the results of the STGCN-based clustering (Clustering STGCN) and the clustering based on the engineered features (Clustering FE) for all data points or frames. Additionally, the distances (Distances) among the robot, the human left and right hands, and the object are contrasted with the embeddings or STGCN output features (Embeddings). Notably, blue colors denote different clusters, but other blue colors are used for possibly similar clusters per clustering approach. Overall, STGCN-based clustering results in subtler distinctions in interactions with the same number of clusters compared to clustering with engineered features. The figure also shows that the embeddings resemble the distance features. This is obvious as the distances were incorporated into the STGCN as node features and edge weights, and the similarity of input features and output features was optimized during model training.

Considering all scenarios and their executions would go beyond the scope of this paper. Thus, the performance of STGCN-based clustering and its differences from the comparison clustering will be discussed in more detail based on the following examples.

**FIGURE 10:**
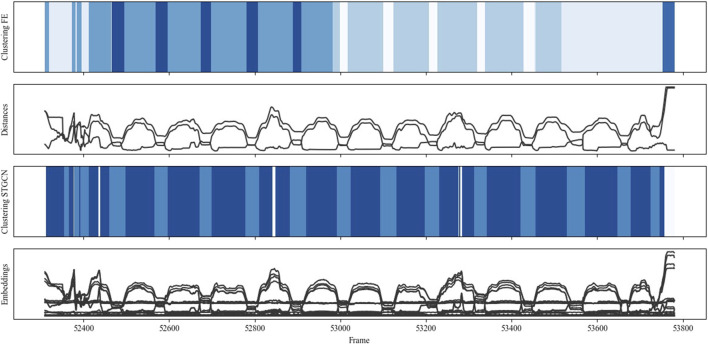
Clustering comparison for scenario I: “pick and place.”

Figure 10 visualizes the data, modeling, and clustering results collected during one run of the pick and place scenario (scenario I). Whereas the 12 picks can be recognized based on the peak patterns from distances and embeddings, as well as the darker blue areas resulting from the STGCN-based clustering, the approaching and indirect overhanding between the human and robot is represented by the valleys and the lighter blue areas from STGCN-based clustering. The results indicate that the STGCN-based clustering can reliably and reproducibly identify different interactions, such as the predominantly coexisting behavior during part retrieval and the more synchronized sequential behavior during indirect handover. In addition, comparison clustering detected most but not all pick and place interactions correctly. Both the peaks and valleys were assigned to two different colors or clusters based on the engineered features. This suggests that the concerned clusters may not differ significantly in terms of the mapped interaction behavior but rather in terms of marginal differences in distances. Furthermore, the three thin, very light blue lines indicate that STGCN-based clustering can reliably detect even subtle behavioral differences, such as when the human is fetching the next frame.

**FIGURE 11:**
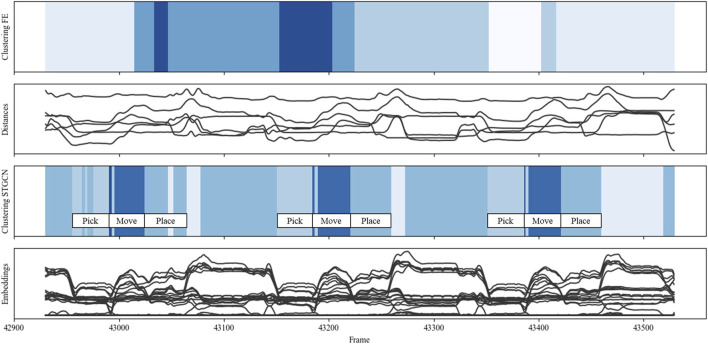
Clustering comparison for scenario B: “co-manipulation.”

The results from data collected during one run of the co-manipulation scenario (scenario B) are shown in Figure 11. Here, the human and robot simultaneously grasp a plank at opposite ends, lift it off a stack, move it, and place it on another stack. As labeled in the figure, STGCN-based clustering reliably recognized the simultaneous pick, move, and place actions from humans and robots. Small fluctuations and differences from the other marked patterns can only be seen in the left-hand marked “pick-move-place” pattern. This indicates that STGCN-based clustering could be further improved in terms of robustness.

In contrast to STGCN-based clustering, no recurring patterns can be recognized in comparison clustering. Only cluster changes in the corresponding segments indicate that interaction changes are perceived. However, these cannot be assigned to any clear clusters.

## 5 Discussion

**FIGURE 12:**
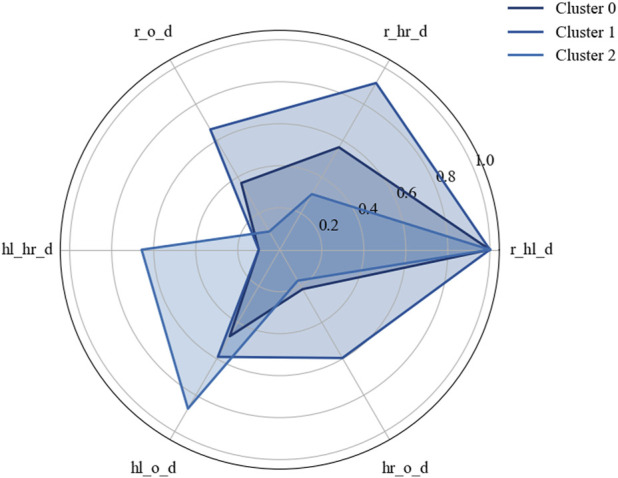
Characterization of some clusters using engineered distance feature mean values.

Both examples showed that the STGCN-based clustering can find meaningful, distinct clusters or interaction forms. A detailed examination, characterization, and description of the identified clusters would exceed the scope of this paper. However, this is planned and can be supported by the statistical analysis of the engineered features from all data points assigned to a cluster and identifying the most important features, such as by using methods from the explainable machine learning field (feature importance, SHAP, *etc*.). A preliminary statistical characterization of the identified clusters was performed using the engineered distance features. Figure 12 visualizes the characteristics of some clusters using the mean values of the distances between the human left and right hands, robot, and object. The results revealed distinct spatial patterns across clusters, supporting their behavioral relevance.

The results indicate that approaches combining spatial and temporal convolutions to learn both spatial and temporal behavior can outperform more classical clustering approaches, and this also applies to industrial human–robot interaction analysis based on tracking data. In addition to others, this supports the findings of Yan et al. (2018), Liu et al. (2024) and Xing and Burschka (2024), who derived graph data from videos and successfully detected human actions with spatiotemporal convolutional network architectures. However, slight variations and mismatches in the STGCN-based clustering results indicate that the approach and model could be further improved in terms of robustness and reliability. These inconsistencies may stem from multiple factors. Minor tracking inaccuracies or latency in the tracking system could introduce small deviations in the recorded trajectories, especially during fast or subtle human movements. As human and robot movements take place in continuous space and the interactions blend into another, sequences may lie near boundaries between interaction forms, such as transitions from passive observation to active collaboration, making them inherently ambiguous. Furthermore, limitations in the current model architecture, such as the fixed temporal window size and the shallow network depth, may constrain the ability to capture long-range dependencies or contextual shifts. Addressing these issues in future work may involve refining the model architecture (e.g., the number of layers, number of channels, and other hyperparameters), incorporating noise-robust preprocessing techniques, or enriching the input features with additional semantic or contextual information.

Usually, the interaction forms’ coexistence, sequential and parallel cooperation, and (responsive) collaboration are distinguished in industrial human–robot co-work (Behrens, 2019; IFR, 2024). For all the tried clustering approaches on the collected interaction data, the metrics consistently suggested a range between eight and 11 clusters, with k = 10 yielding the balance across all indicators (low DBI, high CHI, and a noticeable elbow in the SSE curve). This multi-metric evaluation supports the selection of k = 10 and suggests that human–robot interactions are more diverse than the conventional taxonomy with four interaction forms that are captured. A more granular clustering enables a better reflection of subtle differences in spatial, temporal, and object-centered behavior.

**FIGURE 13:**
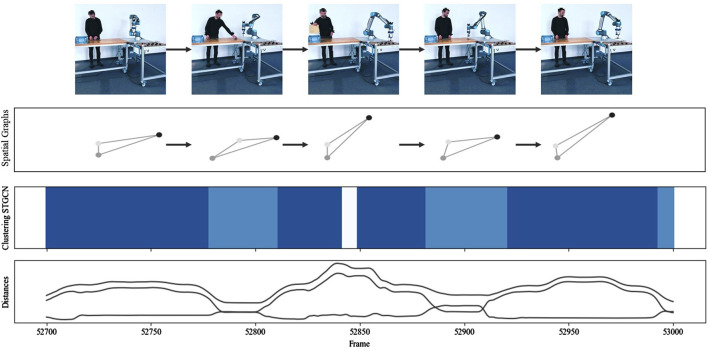
Interaction description for a process section from scenario I “pick and place.”

The presented approach, adapted for classification, if necessary, enables a more automatic, continuous investigation of continuously changing human–robot interactions and more flexible human–robot co-work by reducing the manual adaptation effort. At the top of Figure 13, several video frames show the process sequence carried out during the data collection in scenario I “pick and place.” The collected tracking data can be used to generate a spatial graph for each point in time and calculate the distances among all nodes in a graph. As the picked parts are too small, a tracker would hinder the foreseen object manipulation and change too frequently, so no object tracker was used in this scenario. Consequently, each graph consists of a node representing the robot (black) and nodes representing the human’s left and right hands (grays). In other scenarios, graphs comprised four nodes. The graphs not only help to visualize the spatial interaction behavior in a simplified and structured form but, together with the distance information, also serve as an input for the STGCN model and the subsequent clustering process.

Figure 10 shows that STGCN-based clustering can reliably and reproducibly identify different interactions, such as the predominantly coexisting behavior during part retrieval and the more synchronized sequential behavior during indirect handover. In this case, the various interactions behind the shades of blue were described manually using contextual knowledge. However, it can be partially automated using descriptive statistics and methods from interpretable machine learning.

Whereas the figures above visualize cluster assignments over time along the process and allow the detection of recurring temporal patterns, chord diagrams (or heatmaps) enhance the interpretability of the clustering results and provide a complementary perspective by illustrating the interactions and relationships between clusters.

**FIGURE 14:**
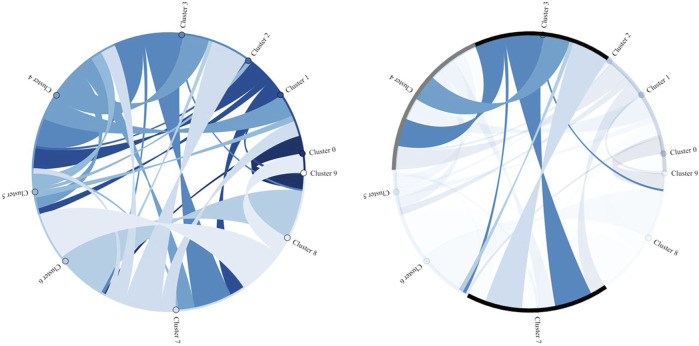
Interaction among all clusters (left) and highlighted interactions of cluster 3 (right).

Figure 14 presents, on the left side, the interaction transitions among all 10 clusters, where the width of each ribbon indicates the transition frequency. This reveals both dominant connections and subtler interdependencies in the data. The right side highlights the interactions of cluster 3, illustrating its strong relationship with clusters 4 and 7.

## 6 Conclusion

In this paper, we present an unsupervised approach for clustering human–robot interaction forms in industrial human–robot co-work scenarios using STGCNs and k-means clustering. The approach is grounded in a novel dataset collected from 12 realistic human–robot co-work scenarios covering various industrial human–robot interactions. A dataset with 1.32 million data points, covering 3.69 h of human–robot interactions, was collected using the capturing approach with the Lighthouse tracking system presented by Heuermann et al. (2024). By modeling the interactions as spatiotemporal graphs and applying a machine learning pipeline, the method enables the automatic detection of nuanced interaction patterns beyond conventional taxonomies.

The results demonstrate that the proposed approach can distinguish 10 clusters—representing 10 interaction forms—in the collected and considered dataset, offering empirical evidence that the spectrum of human–robot interactions is more varied than the typical classification into coexistence, sequential, and parallel cooperation, and collaboration suggests. On the one hand, not all interaction forms are guaranteed to be represented in the scenarios. On the other hand, only those interaction forms that the tracking system can capture are represented in the dataset. The tracking system that was utilized captures high-resolution spatial and motion data but does not record other interaction modalities such as verbal commands, gaze, or cognitive intent. As a result, the dataset may be biased toward physical and spatially measurable behaviors, potentially underrepresenting more abstract forms of human–robot interaction such as verbal supervision, intention signaling, or passive monitoring. Therefore, this list of 10 interaction forms cannot be considered complete. The results suggest that the conventional distinction among four forms is insufficient to describe the interaction behavior at complex production workplaces fully. Future work may mitigate this bias by integrating multimodal sensors or by combining the tracking data with contextual information from task logs, user input devices, or manual behavior annotation. These extensions could provide a more holistic understanding of the full spectrum of human–robot interaction.

Whereas the STGCN model used is based on existing architectures, the contribution of this work lies in integrating established techniques into a structured, data-driven approach for unsupervised interaction clustering in human–robot co-work. This approach enables continuous, data-driven monitoring of interaction behavior and is a basis for more adaptive and intuitive human–robot systems that are aligned with the Industry 5.0 vision. In industrial settings, the proposed approach could be deployed to track how humans and robots interact across workstations in real time. For instance, in assembly lines, it could detect deviations from standard procedures, such as skipped steps or prolonged idle phases, which may indicate fatigue, process bottlenecks, or safety risks. Based on this continuous monitoring, process descriptions and risk assessments could be automatically updated, even as production conditions change dynamically. Additionally, the system could detect evolving interaction patterns over time, enabling robots to adapt their behavior accordingly and support more flexible, context-aware co-work. While the presented approach demonstrates the feasibility and effectiveness of STGCN-based clustering for human–robot interactions, several limitations remain. The results are based on a controlled dataset from 12 predefined scenarios, which cover a wide range but not the entire variability of industrial human–robot co-work. Additionally, only interactions that can be represented by spatial proximity and movement of the tracked entities (human hands, robot, and object) are modeled; more complex behavioral cues are not included. The model currently operates offline, using prerecorded data and batch processing. Real-time online monitoring or prediction deployment would require additional adaptation, including latency optimization and robustness to tracking noise. Finally, the lack of ground truth labels for interaction forms limits quantitative validation. Although unsupervised clustering provides valuable insights, a future step is to annotate parts of the dataset and train semi-supervised or supervised models for benchmarking.

Future work will focus on expanding the dataset to more realistic and varied environments, investigating and improving model robustness (e.g., by conducting a systematic sensitivity analysis), and adapting the approach for online classification and interaction prediction. To achieve this, the current batch-processing architecture would need to be modified for real-time streaming inference, potentially using a sliding temporal window to enable low-latency classification. For online classification, the STGCN architecture would require modification to produce classification outputs, and the model must be trained using labels derived from the unsupervised clustering results. For interaction prediction, a model would need to be trained on the sequences of interaction classes, or alternatively, the STGCN architecture could be adapted for time-series forecasting to predict future interaction states based on past spatiotemporal patterns. Furthermore, integrating explainable and interpretable machine learning techniques can enhance the interpretability of clustering results and support human-centered system design.

## Data Availability

The raw data supporting the conclusions of this article will be made available by the authors, without undue reservation.
